# Associations between lifestyle, erectile dysfunction and healthcare seeking: a population-based study

**DOI:** 10.1080/02813432.2020.1753347

**Published:** 2020-04-21

**Authors:** Louise Herluf Paulsen, Louise Sørensen Bakke, Dorte Ejg Jarbøl, Kirubakaran Balasubramaniam, Dorte Gilså Hansen

**Affiliations:** Research Unit of General Practice, University of Southern Denmark, Odense C, Denmark

**Keywords:** Erectile dysfunction, lifestyle, healthcare seeking, self-report, general practice, general population

## Abstract

**Objective:** To investigate associations between age, lifestyle and erectile dysfunction (ED) in the general population and to explore associations between age, lifestyle and contact with a general practitioner (GP) regarding ED.

**Design:** Cross-sectional web-based questionnaire study.

**Setting:** The general Danish population.

**Subjects:** A randomly selected sample of 48,910 men aged 20 years and older.

**Main outcome measures:** Prevalence of ED and probability of contacting a GP regarding ED. In logistic regression models we analysed associations between age, smoking status, alcohol consumption, body mass index (BMI), and self-rated physical fitness on both ED and GP contact.

**Results:** A total of 22,198 men (47.6%) completed the question regarding ED. The overall prevalence of ED was 19.3%, varying from 2.3% among men aged 20–29 years to 55.3% among men aged 80 years and above. 31.8% of men reporting ED had contacted a GP regarding ED. Increasing age, current or former smoking, complete alcohol abstinence or alcohol consumption above seven units per week, high BMI, and poor self-rated physical fitness were significantly associated with reporting ED. The proportion of GP contacts was significantly associated with age. Overall, no significant associations between lifestyle and healthcare seeking were observed, although lower odds of GP contact were found when physical fitness was rated as poor.

**Conclusion:** Reporting ED and GP contact were significantly associated with age. Furthermore, lifestyle was significantly associated with reporting ED, but largely not associated with healthcare seeking. These findings are important for future interventions aiming to improve diagnosis and treatment of ED.

**Key points**
Experiencing erectile dysfunction is frequent in the general population, especially among older men.
  • In this large-scale national survey, age and lifestyle were significantly associated with reporting erectile dysfunction.
  • Healthcare seeking with erectile dysfunction was significantly associated with age, but not with lifestyle.
  • Diagnosis and treatment of erectile dysfunction might be challenged when erectile dysfunction does not lead to healthcare seeking.

## Introduction

Erectile dysfunction (ED) is a commonly occurring condition among men in the general population and has a negative impact on quality of life and sexual relationships [1,2]. Existing literature reports a large variation in the estimated prevalence of ED, ranging from 5% to 52% [3,4]. This variation can be due to differences in study populations, research methods, and definitions of ED [5]. According to the Fourth International Consultation on Sexual Medicine, ED is defined as the ‘consistent or recurrent inability to attain and/or maintain penile erection sufficient for sexual satisfaction’ [6].

ED can be due to organic (i.e. neurogenic, endocrinological, vasculogenic, or drug-induced), psychogenic, or mixed causes [7]. Additionally, ED and cardiovascular disease (CVD) share many risk factors including advanced age, cigarette smoking, excessive alcohol consumption, a high body mass index (BMI), and sedentary behaviour [1,5,7–10]. The association between these lifestyle factors and ED has mainly been studied in relatively small sample sizes. ED is additionally an independent predictor of cardiovascular events, appearing three to five years prior to an event, and is suggested to be a sentinel marker for subclinical CVD [8,9].

Pharmacological treatment with oral phosphodiesterase type 5 inhibitors is currently the first-line treatment for ED, while statins have a preventive effect on CVD and probably also on ED [7,8]. Lifestyle modifications such as increased physical activity, weight loss, and smoking cessation have also demonstrated a preventive effect on ED and CVD, and may possibly contribute to incremental benefits superior to that achieved with medical treatment for ED [7,8,11,12].

A fundamental requisite for pharmacological treatment and lifestyle intervention in general practice is the individual’s willingness to seek healthcare. Previous studies indicate that only one-third of men with self-reported ED have sought professional help [13,14]. One study found that patients prefer that physicians initiate discussions about sexual concerns [15]. Factors that motivate men with ED to consult a physician include dissatisfaction with sexual life, the severity and duration of ED, and the influence of a spouse or sex partner [13,16,17]. Whether lifestyle influences healthcare seeking remains unclear. One study has shown that certain lifestyle factors influence healthcare seeking with respiratory alarm symptoms (RAS) [18]. Whether this is also the case among men with ED is yet to be determined, but this might be relevant knowledge for future interventions aiming to increase awareness in the general population and among general practitioners (GP) and thereby improve diagnosis and prevention of ED as well as CVD.

Based on a large-scale national survey, this study aims firstly to investigate associations between age, lifestyle, and the prevalence of ED in the general population, and secondly to explore associations between age, lifestyle, and contact with a GP regarding ED.

## Material and methods

### Study design and subjects

This study was a part of the nationwide Danish Symptom Cohort Study (DaSC), which included a cross-sectional web-based questionnaire study. The conceptual objective of the DaSC study was to estimate the prevalence of various symptoms in the general population, including ED, and to determine healthcare seeking behaviour and the individuals’ reaction to each symptom [19].

A total of 100,000 individuals (48,910 males) aged 20 years and older were randomly selected from the general population by use of the Danish Civil Registration System (CRS). In the CRS, all persons with a permanent residence in Denmark are assigned a unique personal identification number [20]. Individuals who had declared that they did not want research-related inquiries were excluded prior to sampling.

The selected individuals received a postal invitation containing a description of the study as well as a 12-digit login for the web-based questionnaire. Non-respondents were sent a reminder letter after two weeks, and after another two weeks, they were contacted by telephone and encouraged to participate. Individuals with no access to a computer were offered the option of completing the questionnaire *via* a telephone interview. The data was collected from June to December 2012.

### The questionnaire

The questionnaire included items covering symptom experiences within the preceding four weeks and healthcare seeking for a wide range of different symptoms [19]. This paper addresses experiences of ED. The question regarding ED was: ‘Have you experienced erectile problems within the past four weeks?’ If erectile problems were reported, the follow-up question was: ‘Have you been in contact with your GP, personally, by telephone or email due to your erectile problems?’ The definition of erectile problems was not further outlined, and it will be referred to as ED in the present study. It was possible to choose ‘do not wish to answer’ to the question regarding ED. In addition to the questions about symptom experiences and healthcare seeking behaviour, the participants reported smoking and alcohol habits, weight, height, and self-rated physical fitness.

Standard rating scales, previously validated questionnaires, and ad hoc terms were used for developing the questionnaire. Pilot and field testing confirmed that the question regarding ED was understandable, acceptable, and used as intended. Further details regarding the study design and the development of the questionnaire have been described in detail in a separate article [19].

### Statistical analyses

Two outcomes of interest were studied: first, the prevalence of ED in all men, and second, the probability of contacting a GP regarding ED. We used logistic regression models to analyse associations between age and lifestyle factors on ED and GP contact, respectively. Age (20–29, 30–39, 40–49, 50–59, 60–69, 70–79, ≥80 years), smoking status (never, former, current), alcohol consumption (0, 1–7, 8–21, ≥22 units/week), BMI (in kg/$m^{2},$ underweight (BMI < 18.5), normal weight (18.5–24.9), overweight (25–29.9), obese (≥30) [21]), and self-rated physical fitness (very good, good, fair, not so good, poor) were all included as covariates. Both uni- and multivariable models were applied, accounting for both age and lifestyle factors. The overall association between the categorical covariates was evaluated with Wald tests. We did not account for multiple testing. All data analyses were conducted using Stata (StataCorp. Stata Statistical Software. College Station, TX: StataCorp LLC). A *p*-value below 0.05 was considered significant.

## Results

**Figure 1.**
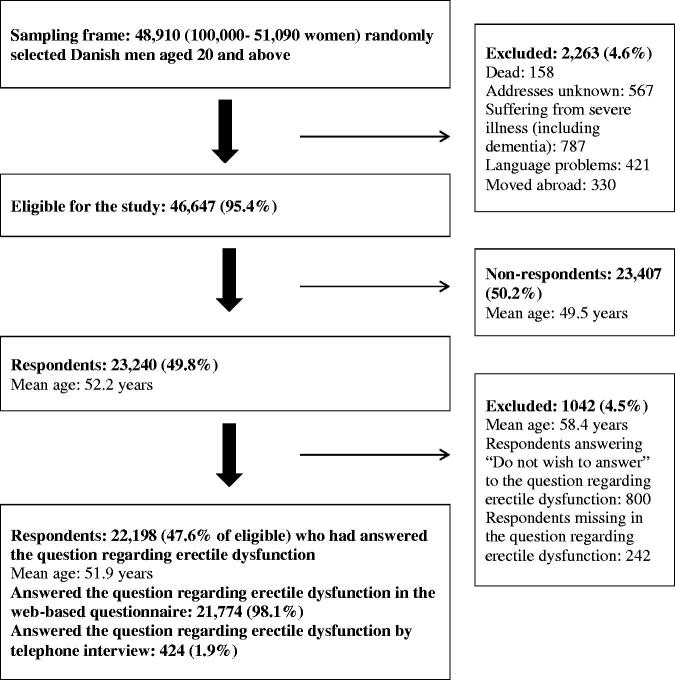
Study **c**ohort.

A total of 48,910 Danish men were invited to participate in the survey. 2263 men (4.6%) were ineligible for the study since they had either died, could not be reached due to unknown addresses, were suffering from severe illness (including dementia), had language barriers, or had moved abroad. A total of 23,240 (49.8%) completed the questionnaire. However, 1042 (4.5% of respondents) were excluded because they either did not wish to answer or were missing in the question regarding ED, resulting in a study cohort of 22,198 men (47.6%) (Figure 1).

A total of 4289 men (19.3%) reported that they had experienced ED within the preceding four weeks, and 1362 (31.8%) had consulted their GP regarding ED (Table 1). The prevalence of ED and the proportion of individuals who contacted a GP are presented by age group, smoking status, alcohol consumption, BMI, and self-rated physical fitness (Table 1).

**Table 1.** Prevalence of ED and proportion of GP contacts regarding ED by age, smoking status, alcohol consumption, BMI, and self-rated physical fitness.

	Total study sample (*n*)	ED, *n* (%)	GP contacts regarding ED, *n* (%)
Total	22,198	4289 (19.3)	1362 (31.8)
Age (years)			
20–29	2289	53 (2.3)	4 (7.6)
30–39	3077	97 (3.2)	27 (27.8)
40–49	4169	271 (6.5)	76 (28.0)
50–59	4706	769 (16.3)	259 (33.7)
60–69	5034	1640 (32.6)	565 (34.5)
70–79	2346	1140 (48.6)	363 (31.8)
≥80	577	319 (55.3)	68 (21.3)
Smoking status			
Never	9129	1155 (12.7)	372 (32.2)
Former	7473	1981 (26.5)	652 (32.9)
Current	5022	989 (19.7)	301 (30.4)
Missing	574	164 (28.6)	37 (22.6)
Alcohol consumption (units/week)			
0	1066	259 (24.3)	93 (35.9)
1–7	12555	2052 (16.3)	663 (32.3)
8–21	6589	1399 (21.2)	444 (31.7)
≥22	1414	415 (29.4)	125 (30.1)
Missing	574	164 (28.6)	37 (22.6)
BMI			
Underweight (<18.5)	112	18 (16.1)	4 (22.2)
Normal (18.5–24.9)	8994	1323 (14.7)	411 (31.1)
Overweight (25–29.9)	9245	1907 (20.6)	630 (33.0)
Obese (≥30)	3225	856 (26.5)	273 (31.9)
Missing	622	185 (29.7)	44 (23.8)
Self-rated physical fitness			
Very good	2001	204 (10.2)	66 (32.4)
Good	9177	1458 (15.9)	482 (33.1)
Fair	7509	1602 (21.3)	505 (31.5)
Not so good	2354	650 (27.6)	221 (34.0)
Poor	583	211 (36.2)	51 (24.2)
Missing	574	164 (28.6)	37 (22.6)

ED: erectile dysfunction; GP: general practitioner; BMI: body mass index.

### Age

The prevalence of ED increased with age, ranging from 2.3% in the age group 20–29 years to 55.3% in the age group ≥80 years (Table 1). The prevalence of ED among men aged 40 years and older was 24.6%. The associations between age and the prevalence of ED were statistically significant, with 50-fold increased odds of reporting ED in the oldest age group (OR = 50.38, 95% CI: 36.45–69.65) in comparison with the youngest age group (Table 2). Higher odds of GP contact were observed with increasing age up to the age group 60–69 years; subsequent odds of GP contact decreased from the age of 70 years and older, although they were still significantly higher when compared with the youngest age group (Table 2).

**Table 2.** Associations between age, lifestyle factors and the prevalence of ED and contact with GP, respectively.

	Prevalence of ED			Contact with GP regarding ED		
	OR (95% CI)	ORadj^a^ (95% CI)	*p*-value	OR (95% CI)	ORadj^a^ (95% CI)	*p*-value
Age (years)			<0.001^c^			<0.001^c^
20–29	1 (Ref)	1 (Ref)		1 (Ref)	1 (Ref)	
30–39	1.37 (0.98–1.93)	1.30 (0.92–1.83)	0.131	4.72 (1.55–14.36)	4.72 (1.54–14.43)	0.007
40–49	2.93 (2.18–3.96)	2.69 (1.99–3.64)	<0.001	4.77 (1.67–13.69)	4.70 (1.63–13.57)	0.004
50–59	8.24 (6.21–10.94)	7.33 (5.51–9.76)	<0.001	6.22 (2.22–17.43)	6.20 (2.20–17.48)	0.001
60–69	20.39 (15.43–26.94)	18.95 (14.29–25.14)	<0.001	6.44 (2.31–17.93)	6.32 (2.25–17.73)	<0.001
70–79	39.88 (30.02–52.99)	40.12 (30.07–53.53)	<0.001	5.72 (2.05–15.98)	5.55 (1.97–15.60)	0.001
≥80	52.16 (37.95–71.69)	50.38 (36.45–69.65)	<0.001	3.32 (1.16–9.52)	3.19 (1.10–9.24)	0.032
Smoking status			<0.001^c^			0.317^c^
Never	1 (Ref)	1 (Ref)		1 (Ref)	1 (Ref)	
Former	2.49 (2.30–2.70)	1.21 (1.11–1.33)	<0.001	1.03 (0.88–1.21)	1.04 (0.89–1.22)	0.605
Current	1.69 (1.54–1.86)	1.34 (1.20–1.49)	<0.001	0.92 (0.77–1.11)	0.93 (0.77–1.12)	0.430
Missing	2.76 (2.28–3.34)	1.51 (0.76–2.99)	0.240	0.61 (0.42–0.90)	0.52 (0.19–1.42)	0.202
Alcohol consumption (units/week)			<0.001^c^			0.194^c^
0	1.64 (1.42–1.90)	1.57 (1.33–1.87)	<0.001	1.17 (0.90–1.54)	1.22 (0.93–1.62)	0.154
1–7	1 (Ref)	1 (Ref)		1 (Ref)	1 (Ref)	
8–21	1.38 (1.28–1.49)	1.11 (1.02–1.21)	0.014	0.97 (0.84–1.13)	0.94 (0.81–1.09)	0.425
≥22	2.13 (1.88–2.41)	1.38 (1.20–1.58)	<0.001	0.90 (0.72–1.14)	0.87 (0.69–1.10)	0.235
Missing	2.05 (1.70–2.47)	1.51 (0.76–2.99)^b^		0.61 (0.42–0.89)	0.52 (0.19–1.42)^b^	
BMI			<0.001^c^			0.922^c^
Underweight (<18.5)	1.11 (0.67–1.84)	0.89 (0.48–1.63)	0.705	0.63 (0.21–1.94)	0.86 (0.27–2.73)	0.792
Normal (18.5–24.9)	1 (Ref)	1 (Ref)		1 (Ref)	1 (Ref)	
Overweight (25–29.9)	1.51 (1.40–1.63)	1.21 (1.11–1.32)	<0.001	1.09 (0.94–1.27)	1.04 (0.89–1.21)	0.639
Obese (≥30)	2.10 (1.90–2.31)	1.47 (1.31–1.65)	<0.001	1.04 (0.86–1.25)	0.96 (0.79–1.17)	0.691
Missing	2.45 (2.05–2.94)	2.08 (1.09–3.99)	0.027	0.69 (0.48–0.99)	1.16 (0.46–2.93)	0.758
Self-rated physical fitness			<0.001^c^			0.081^c^
Very good	0.60 (0.51–0.70)	0.66 (0.56–0.79)	<0.001	0.97 (0.71–1.32)	1.01 (0.73–1.38)	0.959
Good	1 (Ref)	1 (Ref)		1 (Ref)	1 (Ref)	
Fair	1.44 (1.33–1.55)	1.57 (1.44–1.72)	<0.001	0.93 (0.80–1.08)	0.94 (0.80–1.10)	0.428
Not so good	2.02 (1.82–2.25)	2.39 (2.11–2.72)	<0.001	1.04 (0.86–1.27)	1.08 (0.88–1.32)	0.485
Poor	3.00 (2.51–3.59)	2.68 (2.17–3.30)	<0.001	0.65 (0.46–0.90)	0.65 (0.46–0.92)	0.014
Missing	2.12 (1.75–2.56)	1.51 (0.76–2.99)^b^		0.59 (0.40–0.86)	0.52 (0.19–1.42)^b^	

ED: erectile dysfunction, GP: general practitioner, Adj: adjusted, Ref: reference group, BMI: body mass index.

^a^ Adjusted for all variables: age, smoking status, alcohol consumption, BMI and self-rated physical fitness.

^b^ Due to the design of the questionnaire answers missing in smoking status were also missing in alcohol consumption and self-rated physical fitness.

^c^ Result from testing the overall association for the categorical variable.

### Smoking

Reporting ED was more frequent among current smokers (OR = 1.34, 95% CI: 1.20–1.49) and former smokers (OR = 1.21, 95% CI: 1.11–1.33) compared to individuals who had never smoked. No significant associations were found between smoking and GP contact (Table 2).

### Alcohol consumption

Significant associations between alcohol consumption and reporting ED were observed. Compared to those with an intake of 1–7 units/week, individuals with an alcohol intake of 0 (OR = 1.57, 95% CI: 1.33–1.87), 8–21 (OR = 1.11, 95% CI: 1.02–1.21), and ≥22 (OR = 1.38, 95% CI: 1.20–1.58) units/week had significantly higher odds of reporting ED. No significant associations were found between alcohol consumption and GP contact (Table 2).

### Body mass index

The odds of reporting ED were significantly higher among overweight (OR = 1.21, 95% CI: 1.11–1.32) and obese individuals (OR = 1.47, 95% CI: 1.31–1.65) compared to individuals with normal BMI. Furthermore, slightly increased odds of reporting ED were observed in the group of underweight individuals, although this was not statistically significant. No significant associations were found between BMI and GP contact (Table 2).

### Self-rated physical fitness

Statistically significant associations between self-rated physical fitness and reporting ED were observed. The worse the individuals rated their physical fitness the higher their odds of reporting ED. Overall, no significant association between self-rated physical fitness and GP contact was found, although individuals rating their physical fitness as poor had lower odds (OR = 0.65, 95% CI: 0.46–0.92) for GP contact regarding ED compared to those rating their physical fitness as good (Table 2).

## Discussion

### Statement of principal findings

This study underlines that ED is frequent, with an overall prevalence of 19% among men in the general population. Increasing age, current or former smoking, complete alcohol abstinence or alcohol consumption more than seven units per week, high BMI, and poor self-rated physical fitness were all significantly associated with reporting ED. Additionally, GP contact was significantly associated with age. The overall proportion of GP contacts regarding ED was 32%, varying from 8% among men aged 20–29 years to 35% among men aged 60–69 years. Overall, no significant associations between lifestyle and healthcare seeking were observed.

### Strengths and weaknesses of the study

A substantial strength of this population-based study is the large study sample. Moreover, the sample was randomly drawn using the CRS which includes all Danish citizens regardless of age, ethnicity, health, and socioeconomic status and was therefore representative of the general Danish population [22].

The overall response rate among men was 49.8%. The respondents were slightly older compared to the non-respondents (Figure 1). More respondents were married/living together, had a high educational and income level, and were affiliated with the labour market. Other characteristics of respondents versus non-respondents are described in detail elsewhere [23]. Whether individuals’ willingness to participate in the study was related to the overall burden of symptom experience is, however, unknown, and the possibility of other differences cannot be ruled out [23].

A total of 1042 men had either missing answers regarding ED or did not wish to answer the question concerning ED, resulting in a response rate of 47.6% for the ED question, which is comparable to similar studies covering self-reported ED [1,4,24]. Individuals whose ED answers were missing or did not wish to answer the ED question were generally older (Figure 1) and might differ on other parameters as well. Overrepresentation of younger individuals may have resulted in an underestimation of the overall prevalence of ED. This issue is considered minimal due to the small percentage of those with ED answers missing or those unwilling to answer the ED question.

Some individuals might consider ED as an intimate topic. Therefore, the use of a web-based questionnaire is an advantage since it provides anonymity. Participants completing the questionnaire by telephone interview might tend to dismiss their ED compared with the ones completing the web-based questionnaire. However, this possible difference is presumed to have minimal influence on the results since only 1.9% of the respondents completed the question regarding ED by telephone interview (Figure 1).

In the present study we focused on associations between age, certain lifestyle factors, ED, and healthcare seeking. This prioritisation leaves out other factors like chronic disease, drug therapy, and socioeconomic status, all of which might be important when studying ED and healthcare seeking and could be included in future studies.

The question in the survey regarding ED can be understood or interpreted differently depending on the participants’ age, socioeconomic background, cohabitation status, sexual activity, etc., which might lead to differential misclassification. The elderly, men living alone, and/or those without an active sexual life might report ED less often, leading to an underestimation of the prevalence. In terms of experiencing ED there might also be a difference between generations and not only age per se. However, our study was not designed to answer this question. Furthermore, lifestyle factors might be over- or underestimated due to self-reporting, since height generally tends to be over-reported, whereas weight and smoking habits generally tend to be under-reported [25,26]. Since participants were asked to rate their physical fitness, and this was not specified in further detail, some may have rated their physical fitness compared to peers and others over all. Whether this has inflicted the results is not possible to address in the present study. A limited time span of four weeks for experiencing symptoms including ED was applied in an attempt to minimize recall bias. However, some may have recalled episodes of ED that occurred prior to the four weeks, and likewise, others may have forgotten episodes within the time frame. The participants’ sexual activity within the past four weeks may presumably have influenced the perceived relevance of ED, the interpretation of ED, and how well the episodes were remembered. In the question concerning contact with a GP, the time frame was not further specified, which may have resulted in different responses to this question. Health literacy and the ability or willingness to seek healthcare regarding ED might also be influenced by the participants’ socioeconomic background, ethnicity, cohabitation status, comorbidity, etc. Furthermore, perceived importance of erection, duration and severity of ED may have influenced the healthcare seeking behaviour.

### Findings in relation to other studies

A comparison of symptom prevalence in different study populations should be done with caution. The overall prevalence of ED found in this population-based study was 19% in men aged 20 years and older and 25% among men aged 40 years and older. Two population-based studies, Quilter et al. [24] and Lyngdorf et al. [4], observed an overall prevalence of ED of 42% (*n* = 599) and 52% (*n* = 2210), respectively. The higher prevalence of ED reported by Lyngdorf et al. might be due to selection of the study population in primary care and differences in the definition of ED. Furthermore, Lyngdorf et al. did not include a time limit for experiences of ED, while Quilter et al. specified a time frame of 6 months. The four-week time frame in the present study might also explain the lower overall prevalence of ED. Additionally, this study used a yes/no question when asking about ED, whereas the other studies used a scale to rank the experience of ED, which might include more cases of mild ED, resulting in a higher overall prevalence in these studies.

Former or current smoking, high BMI, and poor self-rated physical fitness were all significantly associated with reporting ED. These findings support the associations found in previous studies [4,17,27]. Additionally, we found significant associations between alcohol consumption and reporting ED. Ambiguous findings about associations between alcohol consumption and ED have been reported in the literature. This study found a higher prevalence of ED among individuals reporting complete alcohol abstinence or alcohol consumption of more than eight units/week, which is in line with the study by Haro et al. [17]. Other studies, however, have not been able to establish this association between alcohol consumption and ED [4,28].

Overall, 32% of the men reporting ED in the present study had contacted a GP regarding this problem, which is in accordance with existing literature [13,14]. The individuals aged 20–29 years had the lowest proportion of GP contacts regarding ED. Some studies suggest that younger men are more embarrassed to discuss ED with a healthcare professional or tend to believe that their ED will resolve spontaneously, which could explain the fewer reported GP contacts among the youngest men [13,29]. The degree of concern when experiencing ED has a great influence on whether men seek healthcare. One population-based study found that concerns about having ED peak among men aged 50–59 years [1]. This could correspond to the results from this study where respondents in the 50–69 years age group had the highest proportion of GP contacts. Furthermore, we observed a declining tendency to contact a GP in the age groups 70 years and older. Both decreasing concerns about having ED and a perception of ED being a natural part of aging, as found in a previous study, could explain this declination [13] and might well lead to an underestimation of the prevalence of ED.

Several DaSC studies based on the same study cohort have demonstrated that healthcare seeking differs substantially depending on the type of symptom [18,23,30]. One paper described that the proportion of GP contacts among women reporting pain or bleeding during intercourse was 21.1% and 25.9%, respectively [30]. Another paper reported that 39.6% of individuals reporting at least one respiratory alarm symptom (RAS) had contacted a GP regarding the symptom(s) [18]. The lower tendency to seek healthcare with intimate symptoms like ED and pain or bleeding during intercourse might reflect the fact that these symptoms can be considered as taboo in the general population in comparison with RAS.

The study examining RAS additionally demonstrated that age, smoking status and alcohol consumption were significantly associated with GP contact regarding RAS [18]. Only a few studies have previously investigated the influence of lifestyle on healthcare seeking with ED. One study found that smoking was significantly negatively associated with help seeking [13]. Another study found that men who contacted the GP regarding ED were slightly but significantly more overweight compared to the men who did not contact a GP [31]. We found no significant associations between smoking status, alcohol consumption, BMI and contact with a GP. The overall association between self-rated physical fitness and healthcare seeking was not statistically significant, although individuals rating their physical fitness as poor had lower odds for seeking healthcare. Although poor self-rated physical fitness could be a proxy for poor health literacy, we would expect that other lifestyle factors (current smoking, excessive alcohol consumption and high BMI) could be as well. The fact that certain lifestyle factors were significantly associated with healthcare seeking regarding RAS and generally speaking not with ED might indicate that public awareness of lifestyle as a risk factor differs between different conditions.

### Meaning of the study

The present study found that ED is a frequent symptom among men in the general population. Despite free and equal access to health services in Denmark, only one-third of men reporting ED had been in contact with a GP regarding ED. There might be numerous reasons for not seeking healthcare with ED, including feelings of embarrassment. Diagnosis and treatment with medication and/or lifestyle modification might be challenging if ED does not lead to healthcare seeking.

We found significant associations between age, smoking status, alcohol consumption, BMI, self-rated physical fitness, and reporting ED. Additionally, these factors might be correlated with chronic diseases and multimorbidity, implying frequent contact with a GP. However, we found no significant associations between lifestyle and contact with a GP regarding ED. The findings of this study indicate a need for public awareness aiming to increase healthcare seeking with ED. Additionally, we suggest including questions about erectile function in consultations, especially among the elderly. These initiatives might promote diagnosis and treatment of ED in general practice, and thereby improve sexual health among men in the general population.
